# Chronic Sphincteric and Sexual Symptoms Leading to the Diagnosis of a Tarlov Cyst: A Case Report of Effective Surgical Treatment

**DOI:** 10.7759/cureus.85982

**Published:** 2025-06-14

**Authors:** Abdelali Yahia

**Affiliations:** 1 Neurosurgery, Ouargla Regional University Hospital, Ouargla, DZA

**Keywords:** perineural cyst, sacral radiculopathy, sexual dysfunction, tarlov cyst, urogenital dysfunction

## Abstract

Tarlov cysts, also known as perineural cysts, are cerebrospinal fluid-filled sacs that typically form near the dorsal root ganglion, most commonly at the sacral levels S2 and S3. While most are incidentally identified on imaging, symptomatic cysts, particularly in the lower lumbar and sacral regions, can produce neurological symptoms similar to those of a herniated disc and may have a clinically significant impact on urogenital function.

We present the case of a 38-year-old woman with a long-standing history of chronic pelvic dysfunction characterized by recurrent urinary tract infections, urinary incontinence, and perineal hypoesthesia, all of which significantly impacted her quality of life. Imaging studies revealed a Tarlov cyst involving the right S2 nerve root. Surgical intervention, consisting of cyst decompression and nerve root repair, resulted in substantial symptom improvement.

Although most Tarlov cysts are incidental findings, they can cause debilitating symptoms in select cases. The decision to pursue surgical intervention is complex, yet it can yield remarkable improvements, as demonstrated by our patient’s outcome. However, the literature also highlights the potential for recurrence.

This case underscores the importance of considering Tarlov cysts in patients presenting with unexplained chronic pelvic, urinary, or sexual dysfunction. While surgical intervention may provide significant relief in carefully selected cases, it also emphasizes the necessity for early recognition and timely management.

## Introduction

Tarlov cysts, or perineural cysts, are cerebrospinal fluid (CSF)-filled dilatations that form at the junction between the dorsal root ganglion and the posterior nerve root, most commonly at the S2 and S3 levels [1]. Although frequently asymptomatic and discovered incidentally on spinal imaging, symptomatic Tarlov cysts located in the lower lumbar and sacral regions typically present with back pain, coccygeal pain, lower radicular pain, bowel and bladder dysfunction, leg weakness, and sexual dysfunction [2,3].

Magnetic resonance imaging (MRI) is the modality of choice to identify and characterize these lesions, offering detailed anatomical information about cyst size, location, and nerve root compression. Management strategies range from conservative observation to surgical intervention, depending on the severity of symptoms and the radiological evidence of nerve root involvement.

In this report, we present the case of a 38-year-old woman who suffered from chronic sacral radiculopathy characterized by urogenital and sensory symptoms, ultimately attributed to a sacral Tarlov cyst. Surgical decompression and nerve root repair resulted in substantial clinical improvement, underscoring the importance of an individualized treatment approach in managing symptomatic Tarlov cysts.

## Case presentation

We report the case of a 38-year-old woman with a long-standing history of bladder dysfunction, primarily manifested by recurrent urinary tract infections, several of which required hospitalization due to complications. Over time, she developed persistent urinary leakage, significantly affecting her quality of life and personal hygiene. Additionally, the patient reported chronic perineal hypoesthesia that had severely disrupted her sexual life. Despite being married with two children, she had never experienced sexual satisfaction due to the persistent perineal sensory loss.

**Figure 1:**
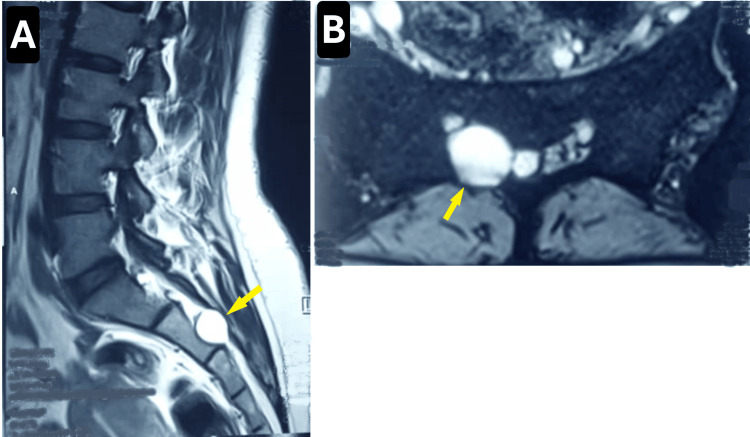
Preoperative MRI findings Sagittal (A) and axial (B) T2-weighted MRI scans show a well-defined, hyperintense, ovoid Tarlov cyst involving the right S2 nerve root, causing compression of adjacent neural structures. Yellow arrows indicate the cyst location.

Lumbosacral MRI revealed a cystic lesion involving the proximal portion of the right second sacral nerve root, consistent with a Tarlov cyst (Figures 1A, 1B).

The strong correlation between her clinical presentation and the imaging findings suggested that her symptoms were primarily attributable to the Tarlov cyst. Consequently, surgical intervention was recommended.

The surgical procedure included fenestration of the cyst wall, decompression of the affected nerve root, and meticulous nerve root repair. The affected sacral nerve root was enveloped with an aponeurotic sleeve and sealed using biological glue to ensure watertight closure and prevent CSF leakage. The postoperative course was uneventful, with no evidence of a CSF fistula.

The patient was subsequently transferred to the physical rehabilitation unit for bladder training, including intermittent self-catheterization to monitor and reduce post-void residual urine.

**Figure 2:**
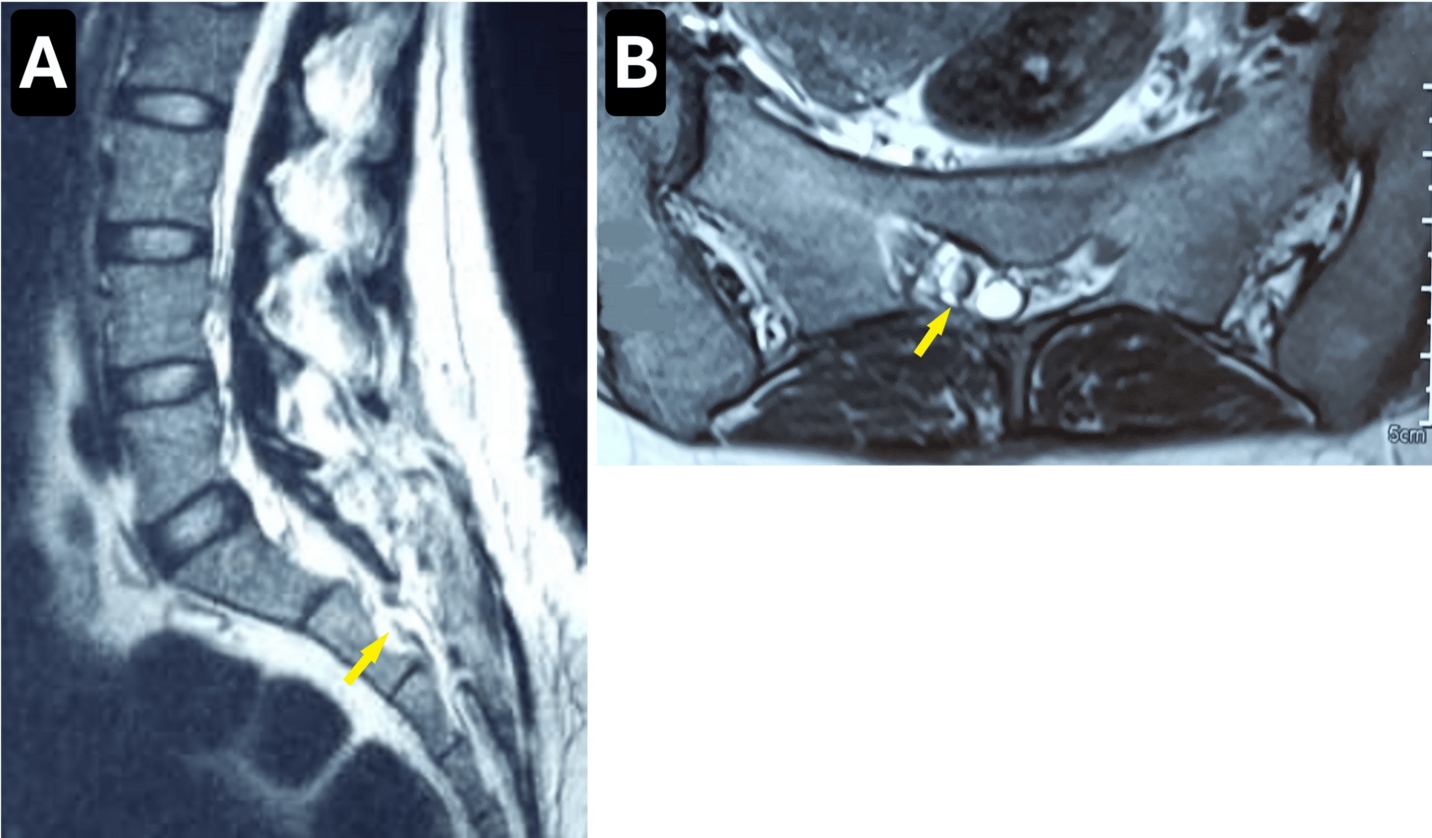
Postoperative MRI findings Sagittal (A) and axial (B) T2-weighted MRI scans obtained six months postoperatively show complete disappearance of the Tarlov cyst, with no evidence of recurrence or residual compression. Yellow arrows mark the previous location of the cyst.

At the six-month follow-up, the patient reported a remarkable improvement in her symptoms. She was able to maintain adequate hygiene between voiding episodes and, for the first time in her life, experienced a fulfilling conjugal relationship, having regained perineal sensitivity and the ability to experience sexual pleasure. A follow-up MRI performed six months postoperatively confirmed complete resolution of the cyst, with no signs of recurrence or other complications (Figures 2A, 2B).

## Discussion

This case highlights the often underrecognized role of symptomatic Tarlov cysts in chronic urogenital dysfunction. Although traditionally regarded as incidental MRI findings, symptomatic Tarlov cysts can cause neurological manifestations such as bladder dysfunction, sexual impairment, and perineal sensory disturbances as demonstrated in our patient [4].

The patient’s prolonged history of urinary incontinence, recurrent urinary tract infections, and perineal hypoesthesia, in the absence of lumbar radiculopathy, initially directed diagnostic efforts toward a purely urological origin. Such presentations often lead to initial diagnostic focus on urological causes. In a study by Baker et al., approximately 50% of women with Tarlov cysts reported urinary urgency and frequency, with many also exhibiting abnormal urodynamic parameters, such as early sensation of bladder filling and detrusor overactivity [5].

The diagnostic shift occurred following lumbosacral MRI, which revealed a cyst involving the right S2 nerve root. In agreement with previously published data, the majority of Tarlov cysts were located at the S2 level, with urinary urgency and frequency being among the most commonly reported symptoms [5]. This case underscores the importance of considering spinal etiologies in patients presenting with otherwise unexplained pelvic or urogenital symptoms.

Surgical treatment of Tarlov cysts remains controversial and technically challenging. However, several studies have demonstrated positive outcomes in carefully selected cases [6,7]. Microsurgical fenestration and nerve root repair, as performed in our case, have been associated with significant symptom resolution. Notably, our patient experienced a dramatic postoperative improvement, not only in urinary control but also in sexual function, resulting in a life-changing outcome, validating the link between her symptoms and the cyst.

The technique employed, combining microsurgical fenestration, dural sleeve reinforcement, and use of biological glue, aligns with strategies described in the literature [8,9]. Importantly, we observed no CSF leakage or postoperative neurological deterioration, complications that have been reported in other surgical series [10].

Minimally invasive approaches, such as CT-guided fibrin sealant injections, have been proposed as safer alternatives and have demonstrated promising results in symptom control with low complication rates [11]. However, for patients with severe and debilitating symptoms and a strong clinico-radiological correlation, surgical decompression remains a valid and often necessary treatment option.

Recurrence of cysts appears to be rare; however, different rates of symptomatic improvement have been reported following microsurgical treatment [3,10]. Notably, nearly half of the patients experienced recurrent pain symptoms [12]. These findings highlight that, although surgery can provide significant benefit in selected cases, outcomes vary and should be carefully considered based on individual patient profiles.

This case reinforces the notion that Tarlov cysts should not be dismissed as mere incidental findings in patients presenting with chronic sphincteric or sexual dysfunction. Instead, a thorough neurological evaluation, including spinal imaging, should be part of the diagnostic workup when conventional urological assessments fail to provide explanations.

Ultimately, further research is needed to refine patient selection criteria, explore the relationship between cyst characteristics (such as size or location) and specific symptom profiles, and establish robust guidelines for the optimal management of symptomatic Tarlov cysts [13].

## Conclusions

This case underscores the importance of considering spinal causes, such as Tarlov cysts, in patients presenting with unexplained chronic pelvic, urinary, or sexual dysfunction. Although often regarded as incidental findings, symptomatic Tarlov cysts can significantly impair quality of life and may warrant surgical intervention when a clear clinico-radiological correlation exists.

Surgical decompression and nerve root repair, as demonstrated in this case, can yield substantial clinical improvement in carefully selected patients. While cyst recurrence is rare, the recurrence of symptoms remains a concern. Increased awareness and early recognition of this condition may facilitate timely diagnosis and more effective management strategies for affected patients.
